# Perception of Medical Undergraduates on Artificial Intelligence in Medical Education: Qualitative Exploration

**DOI:** 10.2196/73798

**Published:** 2025-09-19

**Authors:** Thilanka Seneviratne, Kaumudee Kodikara, Isuru Abeykoon, Wathsala Palpola

**Affiliations:** 1 Department of Pharmacology, Faculty of Medicine, University of Peradeniya, Peradeniya, Sri Lanka; 2 Department of Medical Education, Faculty of Medicine, University of Kelaniya, 4 Thalagolla Road, Ragama, 11010, Sri Lanka, 94 716846317

**Keywords:** medical students, AI in medical education, attitudes, perspectives, learning, qualitative study

## Abstract

**Background:**

Artificial intelligence (AI) has revolutionized medical education by delivering tools that enhance and optimize learning. However, there is limited research on the medical students’ perceptions regarding the effectiveness of AI as a learning tool, particularly in Sri Lanka.

**Objective:**

The study aimed to explore students’ perceived barriers and limitations to using AI for learning as well as their expectations in terms of future use of AI in medical education.

**Methods:**

An exploratory qualitative study was conducted in September 2024, involving focus group discussions with medical students from two major universities in Sri Lanka. Reflexive thematic analysis was used to identify key themes and subthemes emerging from the discussions.

**Results:**

Thirty-eight medical students participated in 5 focus group discussions. The majority of the participants were Sinhalese female students. The perceived benefits included saving time and effort and collecting and summarizing information. However, concerns and limitations centered around inaccuracies of information provided and the negative impacts on critical thinking, social interactions (peer and student teacher), and long-term retention of knowledge. Students were confused about contradictory messages received from educators regarding the use of AI for teaching and learning. However, participants showed an enthusiasm for learning more about the ethical use of AI to enhance learning and indicated that basic AI knowledge should be taught in their undergraduate program.

**Conclusions:**

Participants recognized several benefits of AI-assisted learning but also expressed concerns and limitations requiring further studies for effective integration of AI into medical education. They expressed openness and enthusiasm for using AI while demonstrating confusion and reluctance due to the perspectives and stance of educators. We recommend educating both the educators and learners on the ethical use of AI, enabling a formal integration of AI tools into medical curricula.

## Introduction

As the information age wanes with the rise of artificial intelligence (AI), a global trend has risen to implement AI to enhance the effectiveness of the health care system [1]. Notably, diagnosis and treatments for several diseases can now be performed faster and more precisely with the use of AI in clinical medicine [2,3], giving both doctors and patients easier pathways to navigate diseases than ever before. Consequently, attention is being drawn to how the medical workforce can be ready for this transition and, thus, to investigating how the education of future health care professionals can best be delivered to achieve the futuristic goals of clinical practice [4]. As evidenced by the COVID-19 pandemic, AI poses a significant impact on medical education, with the ability to provide medical students with an interactive learning environment, creating virtual simulations allowing learners to practice complex or risky clinical procedures on virtual patients without endangering actual patients [5-8]. The development of ChatGPT, a new AI-driven language model, showcased its potential for assisting learners in self-directed learning while highlighting ethical issues [9].

The World Medical Association and the Standing Committee of European Doctors advocate for the use of AI systems in basic and continuing medical education [10,11], highlighting the need for increasing awareness of the proper use of AI in graduate, postgraduate, and continuing medical education. However, existing literature on AI emphasizes the inability of today’s medical education to meet the needs of AI, proposing a fundamental change in education to achieve the goals stated by the World Medical Association [2,3,11-13]. An in-depth understanding of how the current medical student perceives the use of AI in their education and their comprehension of limitations, challenges, and future projections is vital for integrating AI into existing medical curricula. Many studies have investigated the students’ perceptions regarding AI in medicine and medical education in countries such as the Republic of Korea [14], Germany [15,16], the United Kingdom [17,18], Canada [19,20], the United States [21], India [22], Pakistan [23], Australia and New Zealand [24], Malaysia [25], Turkey [26], Palestine [27], Saudi Arabia [28], Egypt [29], Syria [30], Jordan [31], the United Arab Emirates [32], and Kuwait [33], and it is worth noting that such examinations are still notably absent in the context of Sri Lanka, a developing island nation in the Asian continent. This scarcity is noteworthy, especially when considering Sri Lanka’s recognized status as a quality health care provider and its contribution to skilled labor migration for developed countries [34]. Recognizing the overwhelming importance of incorporating AI into medical education, this study aims to explore medical students’ perceptions toward AI in education, in terms of the barriers and challenges the students face when using AI in their education, and investigate the future projections of AI into medical education in the eye of the medical student. The findings will aid in making beneficial decisions regarding the inclusion of AI in medical curricula in the future while filling a niche in AI literature on undergraduate medical education.

## Methods

### Study Design

An exploratory qualitative study was conducted in September 2024 using focus group discussions to gather medical undergraduates’ perspectives on using AI in their education. Our objective was to identify barriers and challenges the students face when using AI in their education and to investigate the future projections as perceived by learners to improve their learning experience. We adopted a constructivist approach with the understanding that meaning is constructed through dialog between the researcher and the researched [35]. Semi-structured focus group discussions enabled an in-depth exploration of the student participants’ subjective reality and experiences [36] in using AI in their education and the freedom to consider different issues [37] as the discussions were co-constructed by the researcher and participant [38].

### Ethical Considerations

After obtaining the ethics approval from the Ethics Review Committee, Faculty of Medicine, University of Peradeniya (ERC No: 2024/EC/27), general information about the study was shared with potential participants via WhatsApp groups. Third- and fourth-year medical students of the Faculty of Medicine, University of Peradeniya, and the Faculty of Medicine, University of Kelaniya, were recruited to the study. Students were informed via the information sheet of the voluntary nature of participation, their right to refuse to participate or answer any specific questions, and to withdraw from the study at any time. Informed consent was obtained from all participants. No personally identifiable information was collected, aside from participants’ ethnicity and year of study. All data were securely stored on a password-protected platform, accessible only to the research team. All data collected was anonymized. Purposive sampling was carried out at this stage. To ensure maximal variation sampling, we recruited student volunteers of different genders and ethnic groups, including students from the foreign quota. The interviews were conducted in compliance with the standards for reporting qualitative research set by the Consolidated Criteria for Reporting Qualitative Research (COREQ) [39]. Participants did not receive any incentives for participating in the focus group discussions.

### Designed Focus Group Discussion Protocol

The focus group discussions were facilitated by two trained researchers (KK and WP) in September 2024. The objectives of the present study were explained at the beginning of each focus group, and verbal consent was obtained from each participant. The participants were provided with essential background information. No extra questions were asked after the focus group discussions, no additional information was given by the facilitators, and no other questions were requested subsequently. Only the facilitator and the focus group participants were present for the focus group discussions. Other individuals were not allowed to attend the focus group discussions to ensure the privacy and confidentiality of the discussion. Before recording the responses, permission for audio recording was requested by the facilitator verbally and via the written consent form.

### Data Collection

The participants in the focus group discussions met with a trained research assistant to provide their details and to hand over the consent forms. The time and venue of the focus group discussions were decided at this meeting. We conducted focus group discussions in English using a semi-structured interview guide, which explored how medical students felt about using AI in their education. The questions for the interview guide were extracted from previous similar research [19,27].

We investigated medical students’ perceived barriers and limitations to using AI for learning, as well as their expectations in terms of future use of AI in medical education. The open-ended questions served to guide, but not constrain, the interview. We encouraged the participants to describe their experiences. We emboldened them to react to each other’s opinions and generate new ideas from different points of view. We arranged and conducted focus group discussions, commencing with the first responders. When two successive focus group discussions yielded no novel themes and only repeated ideas, indicating thematic redundancy, we considered that data saturation had been reached [40]. Two trained researchers (KK and WP) conducted all the focus group discussions. Each focus group consisted of 7-9 students. Each focus group discussion lasted 1-1.5 hours and was held at the Faculties of Medicine, University of Peradeniya and University of Kelaniya. With the consent of the students, we audio taped the discussions for later transcription. We informed participants that their identities would remain confidential, and their views and opinions would be anonymized. We removed all identifiable features during transcription. Participants were informed that the focus group discussions constituted part of a research project, and the findings might be published and used to improve medical education.

### Data Analysis

Reflexive thematic analysis (TA) [41-43] was used to analyze the data from the focus group discussions. The qualitative data analysis coincided with the focus group discussions, allowing the researchers to gather information until data saturation. We removed all identifying features during transcription. In analyzing the focus group data, the reflexive element meant that we interpreted the data through our lens of experience as educators, centrally recognizing our subjectivity and seeking to develop a sense of meaning from our responses. As a result, our reflexive analysis of the data led to the output of codes, subthemes, and overarching themes through the coding process [43] and thus represents the reflexive TA approach. This method was structured around the common six-step process for TA as described by Braun and Clarke [41]. Using a sentence-by-sentence process, we manually coded each transcript and sorted the talk into categories and subcategories. All transcripts were coded by two authors (IA and WP). An open coding scheme was used for the first interview. After achieving consensus among the authors, this coding frame was used to code the remaining four transcripts. The authors compared coding for consistency to ensure a common language. We identified commonalities and differences across all interviews before regrouping the codes into themes. We compared interpretations and discussed them among all authors until there were no discrepancies. The authors reached a consensus regarding verbatim remarks selected to highlight the relevant subthemes arising from the analysis.

### Research Team and Reflexivity

Project conception and survey design were performed by TS, who is a consultant pediatrician and a senior lecturer in Pharmacology who believes that AI training in medical education is important and has published similar work. KK is a lecturer in medical education who holds a PhD, with a qualitative and quantitative research background in medical education. Some of the focus group participants were personally known to TS, while all participants were unfamiliar to the second facilitator (KK) and the rest of the research team (IA and WP).

To enhance the credibility of this study, TS, who personally knew some participants and held a senior academic position, refrained from conducting the focus group discussions to minimize the influence of positional authority. KK and WP, to whom the participants were unknown, conducted the focus group discussions. Moreover, member checking on the accuracy of transcriptions was done. All transcripts were coded independently by two authors (IA and WP) who were not directly involved in prior AI-related research. Regular meetings were held to discuss coding decisions and reconcile discrepancies through consensus. We discussed the developed themes and subthemes with KK, an experienced qualitative researcher. TS participated in later stages of theme development, in order to enhance the neutrality and trustworthiness of the analysis. All four authors discussed our own biases to become aware of and be transparent about our perspectives, personal feelings, and preconceptions, and we considered these critically concerning the research being conducted [44]. All authors discussed and resolved any disagreements regarding coding or developing themes. All interpretations were critically reviewed by the entire research team to ensure they were grounded in the raw data. To contribute to the dependability of the data, we kept a reflexivity diary to reflect on the process [45]. We did this because TS is a senior lecturer who had previously contributed to the development of an AI tool used for assessing answers to short answer questions and who believes that AI training in medical education is important. We were cognizant, therefore, that TS may have a propensity to seek the positive elements of the data. In the focus group discussions, KK and WP encouraged participants to express both their positive and negative perceptions, and we consciously sought divergent opinions within the data during analysis. KK and WP emphasized to participants that whatever they mentioned in this study would not affect them in any way in their assessments. To further improve the validity of the findings, all coauthors cross-checked the analysis of all five transcripts.

## Results

### Participants’ Characteristics

In the current study, 38 undergraduate medical students participated in the five focus group discussions. Twenty-two out of 38 (60%) participants were Sinhalese, complying with the composition of the student cohorts in the universities where the study was conducted. Table 1 shows the composition of the focus group discussion participants.

**Table 1.** Composition of focus group discussion participants.

FGD^a^ no	Total number of participants	Gender		Year of study		Ethnicity			
		Male	Female	3	4	Sinhalese	Tamil	Muslim	Other
1	7	4	3	7	0	5	0	2	0
2	8	3	5	0	8	5	1	0	2
3	7	3	4	0	7	5	1	1	0
4	9	4	5	9	0	5	1	2	1
5	7	2	5	7	0	3	0	3	1

a FGD: focus group discussion.

Three major themes emerged from the qualitative analysis: satisfaction with the perceived benefits of using AI for learning, negative attitude toward AI for learning due to perceived limitations, and optimism about the future use of AI to enhance student learning. The themes, subthemes, and initial codes are shown in Table 2.

**Table 2.** Identified themes, subthemes, and codes.

Themes	Subthemes	Codes
Satisfaction with the perceived benefits of using AI^a^ for learning,	Improvement of knowledge	AI for supplementary learning, AI enable identification of knowledge gaps, using AI for problem solving, Using AI for in-depth learning, AI as an enabler of exploration and knowledge expansion, using AI for learning new words/unfamiliar content, AI for preliminary learning, AI for foundational learning.
	Enhanced efficiency	Ease of use of AI tools, using AI is time-saving, AI enables ease of access to information, AI provides streamlined information, AI gives focused answers, AI is useful for organizing information, AI helps directed learning, AI gives quick solutions, AI helps faster reading, using AI requires fewer resources for learning, AI simplifies content.
Negative attitude towards AI due to perceived limitations	Issues with relevance and accuracy	AI gives contradictory information, reliability concerns, no personalization of AI-generated responses, selective applicability of AI, contextual limitations of AI data, AI is fact-centered, AI generates less precise information, AI provides over-generalized information, teachers’ preference for authenticity, need for cross-checking AI information, AI emphasizes minor issues.
	Impact on critical thinking skills	AI promotes surface-level thinking, use of AI causes passive learning, use of AI reduces reasoning ability, use of AI reduces generative ability, AI hinders independent thinking, use of AI reduces creativity.
	Impact on knowledge retention	AI enables short-term memory; using AI reduces long-term memory.
	Impact on collaborative learning and motivation	Use of AI reduces engagement, use of AI reduced peer interactions and fear of social isolation, using AI limits active participation, AI reduces learner motivation for engagement, AI reduces motivation for learning, using AI undermines learner preparation.
Optimism about the future use of AI to enhance student learning	Emerging awareness to guide for better use of AI for learning	AI for objective tasks, selectivity of AI for proper use, not using AI to obtain visual content, use AI for self-evaluation, AI as preparation for lecturer encounters, selective applicability of AI.
	Experiential learning to expand use of AI for learning	Learning from experience, learning AI boundaries, lesser concerns with experience.
	Desire for formal education of AI in curricula	Including AI in teaching, formal teaching of AI, inclusion of AI in curricula, teaching how best to use AI by lecturers.

a AI: artificial intelligence.

### Satisfaction With the Perceived Benefits of Using AI for Learning

The theme of satisfaction with the perceived benefits of using AI for learning developed over many references across all student participants. The participants explained that their motivation to use AI was driven by factors such as simplicity, time-saving qualities, and efficient access to information. The participants unanimously agreed that AI tools improve their knowledge. AI served as a starting point for understanding concepts and generating initial ideas, which participants refined or expanded later using textbooks or other sources:

*AI is a good way to get a basic idea. Once we get that basic idea it is easy to build upon that base by referring to textbooks and other resources.*

[P04]

Participants valued AI for its assistance in answering follow-up questions, clarifying concepts, and exploring unfamiliar theoretical concepts until the students understood thoroughly. As one participant pointed out:

*We can ask the question and get an answer. We can follow up until we understand it. We can ask again and again.*

[P22]

AI helped most students to learn new words and expand their knowledge by identifying gaps or what they may have overlooked when learning or answering questions. Students used ChatGPT to compare the answers and identify missing points:

*Then I look at the ChatGPT answer and realize that I have missed some points, so then I can go back and find these points and add those as well.*

[P03]

All the participants appreciated AI tools for their efficiency and ease of use. AI was seen as an easily accessible, easy-to-use tool that enabled obtaining new ideas and simplified learning, which created an appeal to engage with it for learning. As one participant expressed:

*AI is a very easy method to get an idea about our answers and it’s very interesting. I like to use it.*

[P23]

AI tools were perceived to provide concise, filtered, and summarized information, which provided organized and structured answers to questions. The participants preferred AI tools like ChatGPT over other search engines such as Google because AI was identified to deliver organized and to-the-point answers to their queries:

*Rather than Googling a question, we prefer to use AI...coz we usually get an organized, structured answer to our questions there.*

[P35]

Moreover, all the student participants appreciated the time-saving nature of AI. AI helped direct learners to essential concepts, saving time by offering streamlined information. According to the participants, AI significantly reduced the time required to accomplish tasks such as answering questions:

*It is just simple because it’s time saving when we are writing answers to a theory question.*

[P27]

*It showed you the direction of where you should go when you are writing an answer or when trying to read up something more.*

[P15]

The participants elaborated that the time invested in perusing multiple sources such as videos or scientific reports can now be foregone, with the use of AI that gives the required information much faster than before they used AI for learning:

*ChatGPT gives precise and summarized answers, so it takes less time compared to things like going through Youtube to learn something….No need to refer to many resources to formulate an answer also. Therefore, it is very convenient.*

[P33]

AI was used by almost all the study participants mostly as a shortcut to avoid effort in deeper understanding or problem solving. The quick solutions given by AI with focused answers were viewed as a time-saving method of learning:

*If I have the AI tool I always just look it up and then I get the answer straight away.*

[P19]

### Negative Attitude Toward AI for Learning Due to Perceived Limitations

While all participants confirmed the regular use of AI tools for academic purposes, a recurring element was a lack of certainty regarding the information obtained through AI tools such as ChatGPT. All the participants described a hesitancy to use AI for obtaining factual information, stating concerns over reliability. The students described instances where the information provided by the AI tool differed significantly from textbooks and lecture notes.

*Sometimes the facts they (AI tool) give are different from the facts in lecture notes or reference books.*

[P12]

The participants were discouraged from using AI tools to learn about infections or diseases. The students did not want AI assistance to explain the clinical reasoning processes. The students noted that AI failed to account for geographic, regional, and contextual data, particularly in clinical and epidemiological aspects, and provided contradictory information when compared with trusted sources.

*After a few tries, I have stopped using AI for clinical work. It is not that much fitting to our setting. Mostly I go according to the textbook but sometimes I clear it out with a lecturer.*

[P28]

Some study participants stressed that AI-generated answers sometimes include irrelevant or rare information, which emphasized minor issues, which were viewed as unhelpful and misleading.

*AI gives very minor details at times which are not taught during lectures, and which were not included as objectives in classes.*

[P32]

Hence, the AI outputs were not accepted at face value, and the discussion revealed that students frequently resorted to cross-checking information obtained from AI tools with trusted sources such as Medscape, research articles, or lecture notes to ensure accuracy.

*Most of the time we also cross-check with textbooks or with other articles on the internet like Medscape or research articles.*

[P13]

Moreover, some students were highly dissatisfied with the lack of personalization of AI-generated answers to questions. Students found that the general, less precise, and mechanistic nature of AI-generated information is of lesser value for learning. This feeling intensified as the students felt that AI failed to account for additional details or their individual thought processes, which made students lean toward their teachers to obtain information. As one participant pointed out:

*It is not very personified…might be a bit of a negative thing. Like a teacher would understand what you are trying to say and tell you how to get about it…. but AI can’t recognize that.*

[P04]

Another issue that dissatisfied some students about using AI for their learning was the perceived negative impact on their critical thinking skills. The students were reluctant to use AI as they felt that AI promoted passive learning as it merely encouraged surface-level thinking. Overreliance on AI was felt by students as leading to decreased effort in deeper thought processes, hindering independent thinking, creativity, and reasoning ability:

*Sometimes I feel like we don't think enough, especially during clinicals. Then I get the answer straight away so when I get used to that I stop starting to think about why something is what it is… like that.*

[P07]

*Like it disturbs our thinking ability a lot because you get used to just get it from AI without doing anything of our own. We probably won’t be developing…or doing any thinking of our own.*

[P26]

A few participants were highly worried regarding the perceived impact AI had on their knowledge retention. Students felt that the use of AI promoted only short-term memory and worried that relying on AI would affect their long-term retention as they compared learning from AI with that of peers or their teachers:

*When we are discussing it with others, we feel we can remember it more. If we discuss it with my friends or lecturers, it goes to long-term memory and help to remember things more clearly.*

[P11]

*Sometimes I feel reluctant to use because we don’t think enough. It’s like a flash in the pan.*

[P33]

Another significant barrier to using AI for learning among most medical students was the perceived negative impact on collaborative learning and motivation. Use of AI by students within the classroom for readily obtaining information meant that it undermined the prior preparation of enthusiastic students, which led to demotivation, reduced engagement, and active participation in the classroom.

*We don’t have to prepare and come anymore. It (using AI) actually decreased our participation and motivation.*

[P35]

The participants saw the overreliance on AI as a potential threat to peer interaction and teacher–student relationships, which culminated in fear of social isolation.

*As of now, we still interact with all the students and the lecturers so it hasn’t affected us that much yet… but if we entirely rely on those things only, it will reduce our social interactions it’s highly probable that it will affect us.*

[P29]

Most students were discouraged from using AI tools for their learning by the attitude the teachers showed. As one participant discussed:

*Lectures are very unhappy if we use ChatGPT or anything of the sort. Like, if they feel like we used it for…maybe an essay or whatever, they will shout basically. It’s really easy to just be away from it (AI assistance) than get shouted at you know.*

[P05]

### Optimism About the Future Use of AI to Enhance Student Learning

However, most of the study participants expressed an optimistic view toward the use of AI in the future. This sentiment emerged due to learning from experience that happened over time, which developed an emerging awareness about mitigating issues they encountered before. The students identified the selective applicability of AI, stating various ways in which AI can be used and where AI has failed.

*AI is good to get answers for most MCQs and also for structured answers like one-word answers. But it can’t give pictures… for example, things like that AI can’t give us.*

[P21]

For most of the participants, their initial concerns regarding AI reliability diminished as they gained experience with determining what to ask and how to interpret AI responses.

*Previously we were worried. But it’s not there now. Much. Like we know what should be asked from AI and what should not be asked of AI. It’s good for self-evaluation because we can practice with it.*

[P34]

Some participants of the study used optimism-building to prepare for lecturer encounters, which resulted in clarifying doubts and enabling deeper learning.

*There’s a limit to what you can learn from AI. But if you learn it and then come to a class, then you understand more with what the lecturer says.*

[P01]

The students acknowledged that the age of AI is upon them and acutely felt the need to use AI for academic purposes.

*It’s like you can’t not use it at all right? Everybody use AI for something or other so you would lose if you don’t know how to use it right? So you have to learn one way or another.*

[P13]

Building awareness for better use of AI resulted in a desire among the medical students for formal teaching of the ethical use of AI. Almost all students felt that if the teachers “taught” them how to use it to improve their learning, they would use it better in a more reliable manner. As one participant expressed not so eloquently:

*If they (lecturers) taught us how to use AI properly rather than shouting at us for doing it, I think we would understand how to do it okay. Some of them (lecturers) talk sort of very… I mean look down on us completely, if they feel like we have used it in like in a presentation or anything. Is it such a big deal? Should we not use AI at all?*

[P22]

Most participants of the study felt that formalizing AI learning by various means would facilitate ethical use of AI for both teaching by the teachers and learning by the students.

*Teaching how to give the commands to ChatGPT, and like what can be done and what can’t be done or things like what are the things that are okay to ask from AI… Also like what is okay to use AI for and what not to. Is it ok to get a title or a picture from AI or not? If you get that early on from the lecturers, then we can use it more effectively I think.*

[P09]

## Discussion

### Principal Results

AI has become increasingly integrated into educational settings, offering both opportunities and challenges. This qualitative analysis reveals a complex and nuanced picture of both enthusiasm and concerns. The majority of participating medical students believe that AI is important for enhanced learning and desire to learn more about AI for better use. We also found that despite these attitudes, there remains a reserve for using AI in education brought on by concerns about the reliability, privacy, and ethical issues and a lack of integration of AI into medical education in Sri Lanka. The clinical, scientific, economic, and ethical future of health care will be significantly impacted by AI [46]. The current generation of medical doctors is set to enter an industry that is significantly more AI‐driven than it was when their training commenced. The current study opens the dialog for an inevitable yet successful implementation of AI in medical education.

The participants viewed AI tools as supplementary learning aids, helping students identify knowledge gaps and facilitating problem-solving. Additionally, AI facilitated exploration and aided learning new vocabulary and unfamiliar content, acting as both a preliminary and foundational learning resource. These capabilities align with findings that AI can provide personalized learning experiences, thereby enhancing knowledge acquisition and skill development [47]. The efficiency was identified as another significant benefit of AI tools. This was documented in previous studies where efficiency allowed students to allocate more time to higher-order thinking tasks, thereby enhancing the overall learning experience [48]. The enthusiasm shown by medical students in the global arena is reflected in the current study, who also report limited AI literacy and a wish for formal learning of AI to enhance student learning [15,49,50].

Despite its benefits, study participants were reluctant to use AI for learning mainly due to inaccuracies and contextually irrelevant information that arises from AI. Studies have revealed the necessity of learning how to manage AI-driven misinformation [51,52]. Although AI-assisted learning has been shown to promote active learning and learner engagement [53], the participants of this study perceived that relying on AI would affect long-term retention of knowledge negatively. Moreover, the study demonstrated concerns that reliance on AI may diminish critical thinking abilities. Consistently depending on AI tools has led to deteriorating basic foundational skills, critical thinking, and problem-solving skills [54]. Overdependence on AI tools has been shown to lead to cognitive offloading, where individuals rely on technology for tasks that require analytical thinking, potentially hindering the development of critical thinking skills [47]. This reliance may result in a superficial understanding of content, as students might accept AI-generated information without thorough evaluation.

Contrary to the evidence supporting enhanced collaborative learning in AI platforms [55], the present study reveals the negative impact of AI-assisted learning on collaborative learning. The participants of the study revealed a demotivation to engage with the peers and tutors for learning, due to the ease of accessing required information through AI platforms. Hence, students feared that the use of AI for learning might result in social isolation. However, students were adamant that the envisioned social isolation had only marginally affected them at present. It is important to note that these sentiments could negatively impact collaborative learning experiences and the development of communication skills, which are essential for critical thinking and knowledge application. Moreover, the participants were also concerned about the lack of personalization that human educators provide. Impersonal feedback may not address individual student needs effectively, leading to decreased motivation and engagement in learning activities [47]. These findings of this study demonstrate the importance of equipping medical students as well as educators with the tools necessary to enhance learner engagement in AI-assisted learning platforms [56].

Interestingly, the study participants appeared hopeful regarding the future use of AI to enhance their learning. The students emphasized the importance of emerging and building awareness and experiential learning to expand the use of AI for learning. The students identified the areas where AI tools fail, showing their growing awareness of the boundaries of AI-based assistance. However, they expected formal teaching of how best to use AI for learning by the faculty. This was amidst contradictory messages students received from educators that spanned a ban on incorporating AI into education to embracing AI-assisted learning. In a background where the evidence is overwhelmingly supportive of the ability of AI to enhance student learning [57], the overt reluctance of educators to accept that students are embracing the inevitable is questionable. This attitude may be because of regional factors or due to the traditional teaching/learning methods largely utilized in the education systems in Sri Lanka [58]. This sentiment may also stem from the apparent mistrust the educators may have on the reliability of AI-generated information [59] and doubts regarding students’ misuse of AI tools for assignments and exams, affecting critical thinking and information retrieval skills [60]. Another study revealed that the educators were concerned about students’ self-reliance on AI applications at the cost of traditional teaching methods, which might deprive them of skills best learned in person or group teaching [61]. However, overcoming such reservations is critical to keep abreast of the advancing technologies and ensure that educators facilitate medical students’ learning in the age of technological revolution [62].

A recurring finding in this study was the strong student desire for guidance from faculty on how to use AI effectively for learning. However, this raises an important question: Should all medical educators, including clinicians like surgeons or pediatricians, be expected to teach AI-related content? Given the complex and evolving nature of AI and the already demanding responsibilities of clinical faculty, assigning this task universally may be unrealistic and potentially problematic. The concern is that untrained educators could unintentionally provide inaccurate or conflicting guidance, which was reflected in the confusion expressed by students in our study. Rather than placing this responsibility on individual subject-area faculty, institutions should adopt a more structured and strategic approach. Drawing on Chan’s AI Ecological Education Policy Framework, AI education should be delivered through dedicated units or trained personnel within the institution, ideally through interdisciplinary collaboration. This would ensure consistency, ethical alignment, and adaptability as AI technologies evolve [63]. Formal training and centralized resources for both faculty and students can support responsible AI use while relieving clinical educators of the expectation to independently master and teach AI tools.

While this study briefly referenced ethical concerns related to AI, it is important to more fully acknowledge the complex ethical landscape surrounding its use in medical education. Beyond data privacy and accuracy, there are deeper concerns regarding student development and equity. As Masters outlines in AMEE Guide No. 158, the use of AI tools may inadvertently undermine core competencies in learners, such as critical appraisal, summarization, and ethical decision-making, if overused or uncritically adopted. Furthermore, algorithmic bias, lack of transparency in AI-generated outputs, and passive data surveillance present significant risks to fairness, autonomy, and human dignity in the educational process. These tools can obscure the need for empathy, reflection, and professional judgment—essential traits of a medical practitioner [64].

Similarly, Alam et al argue that medical education must move beyond basic AI literacy to include a critical understanding of the ethical implications of AI-assisted learning and publishing. This includes interrogating how AI might alter authorship norms, contribute to inequities in access and performance, or even reinforce existing systemic biases if not carefully regulated [65]. The path forward, therefore, requires not just training students to use AI responsibly but also preparing institutions to adopt clear ethical guidelines and foster a culture of reflective, values-based technology use. As AI becomes increasingly integrated into medical education and health care, ethical fluency must be considered as essential as digital literacy for both students and educators.

### Limitations

The present study has some limitations. The study did not quantitatively evaluate the impact of AI-assisted learning on achieving learning outcomes, satisfaction, or other measurable aspects of medical education, which could supplement the qualitative findings of this study. Additionally, since the study’s focus was on understanding the perception of students, the perspectives of other stakeholders, such as the faculty and health care professionals, were not captured, and this could be explored in future research.

### Conclusions

In a region where integrating AI into medical curricula is lacking, this study adds to our understanding of what medical students think about the challenges of AI tools in medical education. The present study highlights the improvement of knowledge and enhanced efficiency as the two primary advantages of AI-assisted learning as perceived by medical students. However, several concerns were interwoven within the widespread adoption of AI among the study participants. The reliance on AI was felt to have a negative impact on critical thinking and cognitive engagement, as students prioritized convenience over deep learning. Interestingly, students feared social isolation as a possible future impact of integrating AI into their learning. Students’ lack of understanding of how much AI assistance is accepted at an institutional level and the need to “play” according to teacher preferences confused and hindered the students despite their enthusiasm to use AI for learning. A balanced approach is warranted to maximize the benefits of AI in education while mitigating its limitations. AI should complement, rather than replace, traditional learning methods, with educators guiding students on how to use AI effectively. Future research to explore the contextual factors and policy efforts is critical to refine AI’s role in education, ensuring it enhances learning outcomes while preserving essential cognitive and social development.

## Abbreviations

AI: artificial intelligence
COREQ: Consolidated Criteria for Reporting Qualitative Research
TA: thematic analysis
